# *Gynura divaricata* exerts hypoglycemic effects by regulating the PI3K/AKT signaling pathway and fatty acid metabolism signaling pathway

**DOI:** 10.1038/s41387-020-00134-z

**Published:** 2020-08-14

**Authors:** Wenjun Xu, Zhongxia Lu, Xin Wang, Man Hei Cheung, Meiai Lin, Changyu Li, Yu Dong, Chun Liang, Yitao Chen

**Affiliations:** 1 College of Life Science, Zhejiang Chinese Medical University, 310053 Hangzhou, China; 2 School of Medicine and Pharmacy, Ocean University of China, 266003 QingDao, China; 3 Division of Life Science, Center for Cancer Research and State Key Lab for Molecular Neural Science, The Hong Kong University of Science and Technology, Hong Kong, China; 4 The Second Clinical Medical College, Zhejiang Chinese Medical University, 310053 Hangzhou, China; 5 Zhejiang Academy of Traditional Chinese Medicine, 310053 Hangzhou, China; 6 EnKang Pharmaceuticals (Guangzhou), Ltd, Guangzhou, China

**Keywords:** Fat metabolism, Lipoproteins

## Abstract

**Objectives:**

The study aimed to examine the anti-diabetic effects of *Gynura divaricata* (GD) and the underlying mechanism.

**Methods:**

Information about the chemical compositions of GD was obtained from extensive literature reports. Potential target genes were predicted using PharmMapper and analyzed using Kyoto Encyclopedia of Genes and Genomes (KEGG) and Gene Ontology (GO). To validate the results from bioinformatics analyses, an aqueous extract of GD was administered to type 2 diabetic rats established by feeding a high-fat and high-sugar diet followed by STZ injection. Key proteins of the PI3K/AKT signaling pathway and fatty acid metabolism signaling pathway were investigated by immunoblotting.

**Results:**

The blood glucose of the rats in the GD treatment group was significantly reduced compared with the model group without treatment. GD also showed activities in reducing the levels of alanine aminotransferase (ALT), aspartate aminotransferase (AST), blood urea nitrogen (BUN), and creatinine (CREA). The levels of urine sugar (U-GLU) and urine creatinine (U-CREA) were also lowered after treatment with GD. Bioinformatics analysis showed that some pathways including metabolic pathways, insulin resistance, insulin signaling pathway, PPAR signaling pathway, bile secretion, purine metabolism, etc. may be regulated by GD. Furthermore, GD significantly increased the protein expression levels of PKM1/2, p-AKT, PI3K p85, and GLUT4 in the rat liver. In addition, the expression levels of key proteins in the fatty acid metabolism signaling pathway including AMPK, p-AMPK, PPARα, and CPT1α were significantly upregulated. The anti-apoptotic protein BCL-2/BAX expression ratio in rats was significantly upregulated after GD intervention. These results were consistent with the bioinformatics analysis results.

**Conclusions:**

Our study suggests that GD can exert hypoglycemic effects in vivo by regulating the genes at the key nodes of the PI3K/AKT signaling pathway and fatty acid metabolism signaling pathway.

## Introduction

Diabetes mellitus (DM) is a complex chronic disease affecting >425 million people worldwide^1,2^, with 114 million in China^3,4^. Due to the drastic increase in diabetes prevalence rate, diabetes has become a serious health issue in China.

*Gynura divaricata* (GD), a perennial herb, has been used in treating diabetes, hypertension, and hyperlipidemia^5–7^ in China. Numerous pharmacological studies have shown that extracts of GD can significantly decrease blood glucose levels, improve lipid metabolism, and promote islet β-cell repair^8,9^. Chemically active ingredients such as flavonoids, polysaccharides, and alkaloids have previously been identified in GD extracts^10,11^. However, precise anti-diabetic mechanisms of GD still need to be elucidated.

In the present study, through network pharmacology analysis, we have chosen the phosphoinositide-3 kinase (PI3K)/AKT signaling pathway and fatty acid metabolism signaling pathway as potential targets of GD. Type 2 diabetic model rats established by feeding a high-fat and high-sugar diet followed by streptozocin (STZ) injection, were treated with GD aqueous extracts to study its anti-diabetic effects. Furthermore, the key proteins of the PI3K/AKT signaling pathway and fatty acid metabolism signaling pathway including PKM1/2, P-AKT, PI3K p85, glucose transporter type 4 (GLUT4), AMPK, p-AMPK, peroxisome proliferator-activated receptor-α (PPARα), CPT1α, BCL-2, and BAX in the rat liver were also investigated.

## Materials and methods

### Active chemical compounds of GD and potential target prediction

**Fig. 1:**
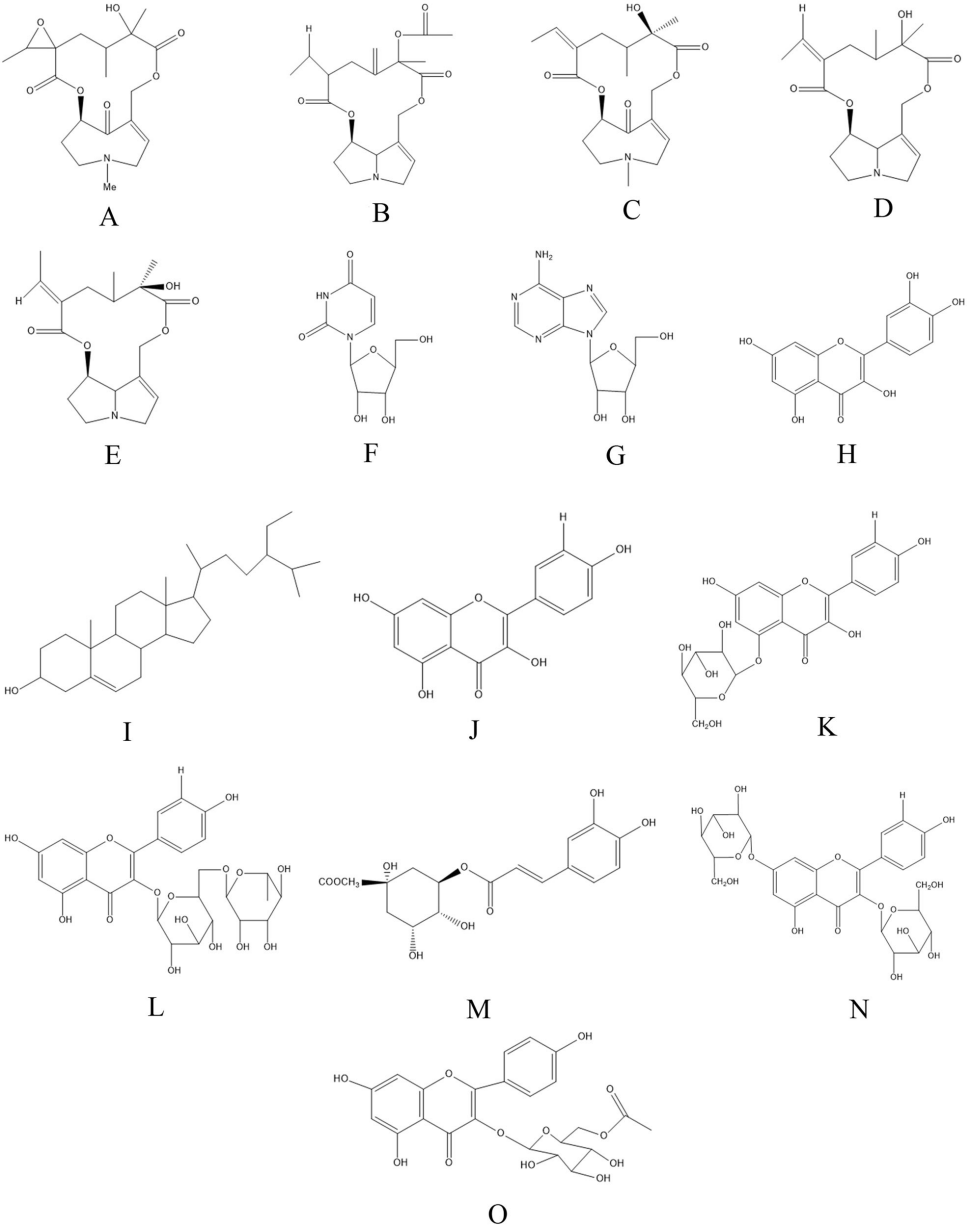
Molecular structures of the 15 major active components in GD. **a** Otosenine (C_19_H_27_NO_7_); **b** sineciphyllinine (C_20_H_25_NO_6_); **c** senkirkine (C_19_H_27_NO_6_); **d** jacobine (C_18_H_25_NO_5_); **e** integerrimine (C_18_H_25_NO_5_); **f** uridine (C_9_H_12_N_2_O_6_); **g** adenosine (C_10_H_13_N_5_O_4_); **h** quercetin (C_15_H_10_O_7_); **i** β-sitosterol (C_29_H_50_O); **j** kaempferol (C_15_H_10_O_6_); **k** astragalin (C_21_H_20_O_11_); **l** nicotiflorin (kaempferol-3-*O*-rutinoside) (C_27_H_30_O_15_); **m** methyl chlorogenate (C_17_H_20_O_9_); **n** kaempferol-3,7-di-*O*-p-d-glucoside (C_27_H_30_O_16_); **o** kaempferol-5-*O*-(6′′-*O*-acetyl)-β-d-glucoside (C_23_H_22_O_11_).

Molecular structures of the 15 components of GD were drawn with the ChemBioDraw Ultra 14.0 software (Fig. 1) and their three-dimensional structures were constructed using the ChemBio3D Ultra 14.0 software. Potential drug targets of the compounds were then searched in the PharmMapper database (http://lilab.ecust.edu.cn/pharmmapper/)^11^. The UniProtKB search function (http://www.uniprot.org/) in the UniProt database was employed to correct the human-related target code to the official names^12,13^. Ten targets with the highest matching scores were selected for each of the compounds. Finally, 40 genes were predicted from 150 genes as potential targets for further investigation after removing duplicated and unannotated genes through bioinformatics analysis.

### Gene Ontology (GO) enrichment and Kyoto Encyclopedia of Genes and Genomes (KEGG) analysis

Information of the potential target proteins was subjected to GO enrichment and KEGG pathway annotation analysis using the Database for Annotation, Visualization and Integrated Discovery (DAVID, https://david.ncifcrf.gov/). Potential target genes of the GD compounds were mapped into the KEGG pathway, and the pathways with a *P* value of < 0.05 were considered reliable.

### GD extraction and identification

GD grown in an environment with relative humidity of 40–60% and temperature of 25 ± 2 °C was purchased from Changxing Pharmaceutical Co., Ltd. (Huzhou, Zhejiang, China). After the plants (including stems and leaves) were crushed, the GD powder was decocted with 1 L of water for 1 h followed by filtration with 8 layers of gauze. The crude drug extract (0.5 g/mL) was concentrated to 1 g/mL by rotary evaporation at 60 °C, 80 rpm and stored at −20 °C for subsequent use.

Some of 50 µL aqueous extract (1 g/mL) of GD were added to a 1.5-mL bullet followed by addition of 950 µL of 50% methanol solution, vortexing for 30 s, sonication for 5 min, and centrifugation for 20 min at 14,000 r/min. Supernatant was taken to obtain sample solutions at a concentration of 50 mg/mL for ultra-high-performance liquid chromatography quadrupole time-of-flight mass spectrometry (UHPLC-Q/TOF-MS) analysis.

### Chromatographic conditions

The chromatographic column used was ACQUITY UPLC BEH C18 (100 × 2.1 mm, 1.7 µm). The mobile phase was 0.1% formic acid in acetonitrile (A)–0.1% formic acid in water (B). The gradient elution procedure was 0–14 min, 99–50% B; 14–16 min, 50–25% B. The volume of each injection was 2 µL and the volume flow rate was 0.3 mL/min. The sample tray temperature was 8 °C and the column temperature was 40 °C.

### Mass spectrum conditions

TurboIon Spray ion source and electrospray ionization positive and negative ion scanning modes were used in TOF-MS. The specific conditions were as follows: Ion Source Gas1 (Gas1): 45 psi, Ion Source Gas2 (Gas2): 35 psi, Curtain gas (CUR): 35 psi, Source temperature: 600 °C, IonSpray Voltage Floating: 5500 V/−4500 V; TOF MS scan *m*/*z* range: 50–1500 Da, production scan *m*/*z* range: 25–1500 Da, TOF-MS scan accumulation time 0.25 s/spectra, product ion scan accumulation time 0.035 s/spectra; Information Dependent Acquisition (IDA) and high-sensitivity mode were used in secondary mass spectrometry. Declustering potential was ±60 V (positive and negative ion modes). Collision energy was 35 ± 15 eV. IDA was set as follows: Exclude isotopes within 4 Da, and candidate ions to Monitor per Cycle were 12.

### SCIEX OS software

SCIEX OS software contains multiple confidence criteria, including quality accuracy, retention time, isotopes, and matching use of compound libraries. In this work, the target compounds were identified by matching with the TCM MS/MS Library (containing secondary data of >1000 Chinese herbal medicines) according to the first-order accurate mass number, isotope distribution ratio, and MS/MS of the compounds.

### Animals

Fifty male Sprague-Dawley (SD) rats (5 rats in each cage, 200 g average body weight) were purchased from the Laboratory Animal Centre of Zhejiang Chinese Medical University (Zhejiang, China). Animal experiments were performed in the Laboratory Animal Centre of Zhejiang Chinese Medical University (rodent license SYXK 2018-0012). This study was approved by the Ethics Committee of Zhejiang Chinese Medical University.

Rats (11 weeks old, 400 g average body weight) were fed with a high-fat and high-sugar diet (Jiangsu Xietong Medicine Bioengineering Co., Jiangsu, China) for 3 weeks and then injected with 30 mg/kg STZ solution (0.1 mM, pH 4.2) through the tail vein to establish type 2 DM (T2DM) model rats. Blood collection was performed with micro-hematocrit capillary tubes (Cas. No. 41B2501; Beijing Dongling Technology Co., Ltd.) through the rats’ ocular vein. One week later, rats with blood glucose level of 15–25 mmol/L were selected as type 2 diabetes model rats, which were randomly divided into 5 groups, namely, model group (DM, administered with water, as a negative control), metformin group (administered with 200 mg/kg metformin, as a positive control), GD low-dose group (administered with 0.5 g/kg GD extracts), GD mid-dose group (administered with 1.0 g/kg GD extracts), and GD high-dose group (administered with 2.0 g/kg GD extracts). Another 10 male SD rats (normal group, treated with water) were fed with a basic diet (Jiangsu Xietong Medicine Bioengineering Co., Jiangsu, China). Carbon dioxide killing box (Cas. No. CL-1000-S2; Shanghai Yuyan Scientific Instrument Co., Ltd.) was used to sacrifice mice, before the end of experiments.

Fasting plasma glucose (FPG) level of the rats was measured by using a handheld blood glucose meter. Blood biochemical indicators, including alanine aminotransferase (ALT), aspartate aminotransferase (AST), blood urea nitrogen (BUN) and glucose (GLU), were measured by a fully automated instrument. Before the end of the experiments, each rat was maintained in a cage for 24 h to record food intake, water intake and urine volume. Urine of the rats in each group was collected for testing urine biochemical indicators. Urine sugar (U-GLU) and urine creatinine (U-CREA) were measured by an automatic biochemical analyzer. The 24-h U-GLU excretion and serum creatinine (CREA) clearance rate were calculated.

### Immunoblotting

Proteins in the insulin signaling pathway, PKM1/2, p-AKT, PI3K p85, GLUT4, AMPK, p-AMPK, PPARα, CPT1α, BCL-2, and BAX, were examined by immunoblotting. At the end of the experiments, the rats were sacrificed with CO_2_ suffocation, and the livers were kept in liquid nitrogen for subsequent experiments. Total proteins were extracted from the livers by incubating with lysis buffer (0.1 mL/g of liver) for 30 min on ice, with agitation every 10 min. After centrifugation for 15 min at 10,000 × *g* at 4 °C, the supernatant was collected, and the protein concentration was determined using the BCA Protein Assay Kit (cat no. KGP902; Nanjing KeyGen Biotech Co., Ltd., Nanjing, China).

Proteins (40 μg/lane) were resolved in 12% sodium dodecyl sulfate-polyacrylamide gel electrophoresis gels and transferred onto polyvinylidene difluoride membrane (Immobilon-FL membrane, Millipore Company, MA, USA). After blocking with 5% milk for 2 h at 37 °C, membranes were incubated overnight at 4 °C with primary antibodies against glyceraldehyde 3-phosphate dehydrogenase, PI3K, p-AKT, PKM1/2, and GLUT4 (#5174, #4257 S, #4060, #3106, and #2213, respectively, at 1:1000 dilution; Cell Signaling Technology, America). The blot was then incubated with horseradish peroxidase-conjugated secondary antibody (A0208 and A0216; Beyotime Biotechnology Co., Ltd, Shanghai, China) for 2 h at 37 °C. Membranes were visualized with a gel documentation system (Aplegen Omega Lum G, American) after incubating with ECL substrate solution for 10 s (P0018A; Beyotime Biotechnology Co., Ltd, Shanghai, China). The signal intensities were quantified using the ImageJ software (version 1.8.0; National Institutes of Health, Bethesda, MA, USA).

### Statistical analysis

All experiments were performed in triplicates. Results were presented as mean ± standard deviation (SD) and statistically analyzed using SPSS 16.0. Results of the fasting blooding level, metabolism, blood biochemistry, and urine biochemistry were subjected to one-way analysis of variance (ANVOA). Immunoblotting results were analyzed by ANVOA. Data with *P* < 0.05 were considered statistically significant.

## Results

### Screening of the active constituents of GD

The diabetes-related active ingredients were determined by their abundance and a large number of reported literature. The molecular structures of the 15 compounds are shown in Fig. 1.

### GO enrichment and KEGG pathway analysis

The PharmMapper database was utilized to predict the potential target genes of the GD active ingredients. Ten genes with the highest matching scores were selected as potential target genes of each compound, and we obtained a total of 150 genes. Finally, 40 candidate anti-diabetes targets were obtained after removing duplicated and unannotated genes through bioinformatics analysis.

**Fig. 2:**
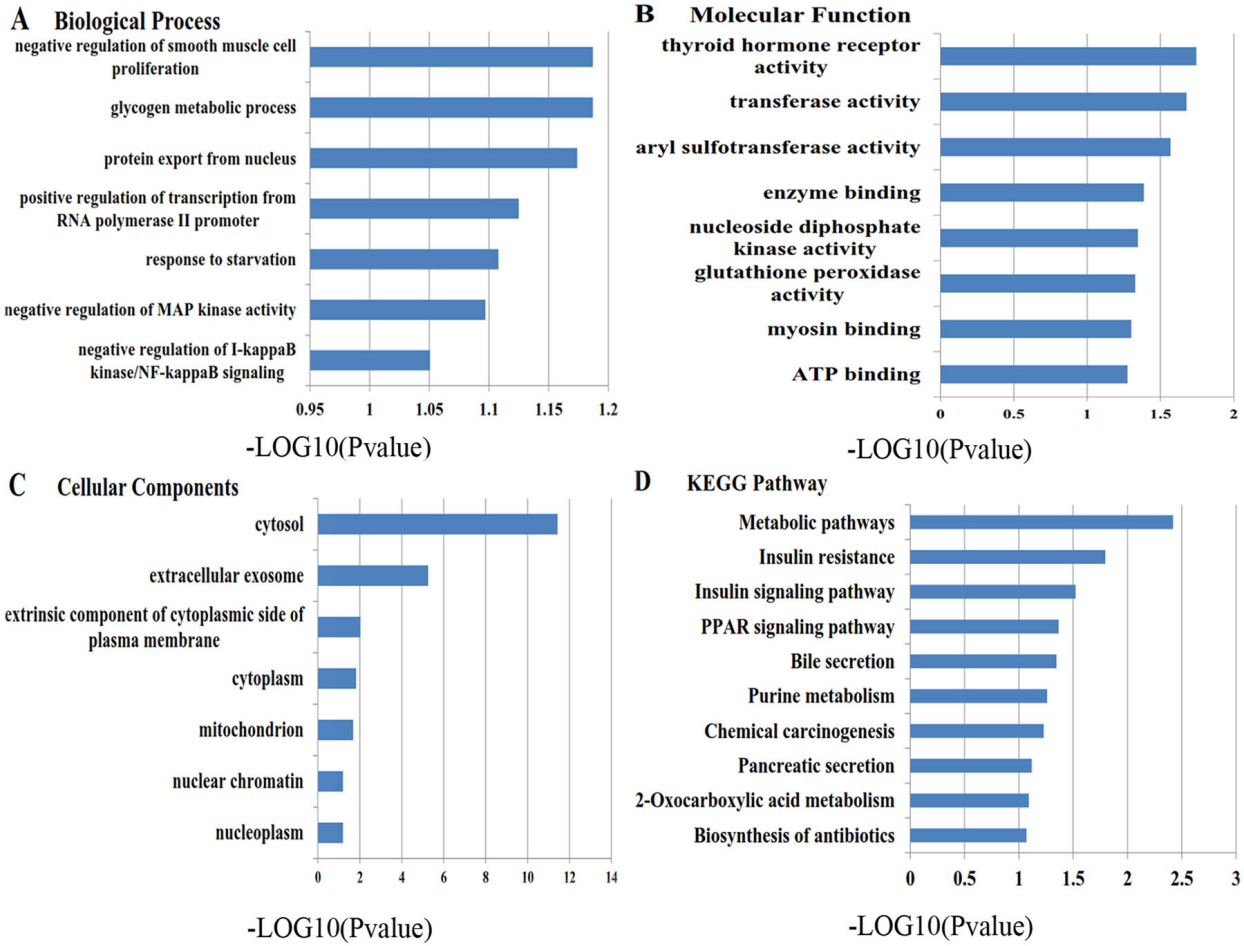
GO enrichment of the GD potential target genes and KEGG pathway analysis results. **a** Biological process; **b** molecular function; **c** cellular component; **d** KEGG pathway.

GO enrichment and KEGG analysis were performed to analyze these genes (Fig. 2). Cellular components affected by GD mainly include extracellular exosome, cytosol, and cytoplasm. For biological processes, GD may affect fatty acid homeostasis, glutathione metabolic process, and cellular response to insulin. Molecular functions including sulfotransferase activity, retinoic acid receptor (RXR) activity, and transferase activity were the main targets of GD. Pathways including metabolic pathways, insulin resistance, insulin signaling pathway, PPAR signaling pathway, bile secretion, purine metabolism, etc. are regulated by GD, according to KEGG pathway analysis. Pathways with a *P* value < 0.05 were considered reliable. Based on these considerations, we chose the PI3K/AKT signaling pathway and fatty acid metabolism signaling pathway, which include PKM1/2, p-AKT, PI3K p85, GLUT4, AMPK, p-AMPK, PPARα, CPT1α, and other proteins, for further analysis and experimental verification.

### Composition analysis of GD

**Fig. 3:**
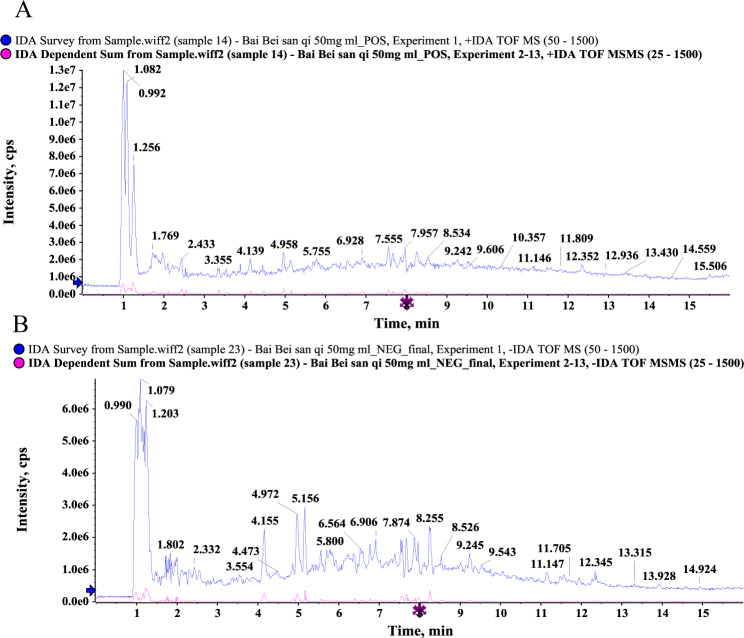
The total ion chromatogram of GD by UHPLC-Q/TOF-MS. **a** Positive ion mode; **b** negative ion mode.

The GD sample solution was analyzed by UHPLC-Q/TOF-MS system, and the total ion flow diagram was obtained. By comparing and screening with the TCM MS/MS Library in the SCIEX OS software, the compounds were identified qualitatively. The identification results are shown in Fig. 3 and Tables 1 and 2. Some 28 compounds were identified under the positive ion mode and 10 compounds under the negative ion mode. Most of these compounds were flavonoids, and it could be inferred that the main hypoglycemic components in GD are flavonoids. The UHPLC-Q/TOF-MS results showed that ten of these compounds are identical to those obtained from network pharmacology shown in Tables 1 and 2, which validated the bioinformatics analysis results.

**Table 1:** High-resolution mass spectrometric data and elemental composition of GD (positive ion mode).

No.	Component name	Retention time	Found at mass	Mass error (ppm)	Formula
1	Arginine	1.13	175.1188	−1.1	C_6_H_14_N_4_O_2_
2	Glutamic acid	1.18	148.0604	−0.1	C_5_H_9_NO4
3	Betaine	1.19	118.086	−1.9	C_5_H_11_NO_2_
4	Proline	1.23	116.0703	−2.9	C_5_H_9_NO_2_
5	Stachydrine	1.26	144.1017	−1.8	C_7_H_13_NO_2_
6	Nicotinic acid	1.72	124.039	−2.4	C_6_H_5_NO_2_
7	Nicotinamide	1.82	123.0551	−1.9	C_6_H_6_N_2_O
8	6-Hydroxypurine	1.87	137.0457	−0.9	C_5_H_4_N_4_O
9	Uridine	2.23	245.0765	−1.2	C_9_H_12_N_2_O_6_
10	Isoleucine	2.43	132.1017	−1.4	C_6_H_13_NO_2_
11	Adenine	2.44	136.0615	−1.7	C_5_H_5_N_5_
12	Adenosine	2.44	268.1036	−1.7	C_10_H_13_N_5_O_4_
13	Cordycepin	2.51	252.1089	−0.8	C_10_H_13_N_5_O_3_
14	Guanosine	2.55	284.0989	−0.2	C_10_H_13_N_5_O_5_
15	Thymine	3.22	127.05	−1.7	C_5_H_6_N_2_O_2_
16	Phenylalanine	3.36	166.0859	−2.2	C_9_H_11_NO_2_
17	Otosenine	4.78	382.1859	−0.2	C_19_H_27_NO_7_
18	Chlorogenic acid	4.96	355.1019	−1.3	C_16_H_18_O_9_
19	Kaempferol-3,7-di-*O*-β-d-glucoside	5.33	611.1605	−0.2	C_27_H_30_O_16_
20	Esculetin	5.47	179.0337	−1.3	C_9_H_6_O_4_
21	Vitamin B2	5.77	377.1452	−0.9	C_17_H_20_N_4_O_6_
22	Loganic acid	5.77	377.145	2.1	C_16_H_24_O_10_
23	Methyl chlorogenate	6.3	369.118	0	C_17_H_20_O_9_
24	Kaempferol-3-*O*-rutinoside	7.54	595.1652	−0.9	C_27_H_30_O_15_
25	Dicaffeoylquinic acid (Cynarin)	7.67	517.1334	−1.2	C_25_H_24_O_12_
26	Astragalin	7.86	449.1076	−0.6	C_21_H_20_O_11_
27	Kaempferol	7.86	287.0547	−1.3	C_15_H_10_O_6_
28	Integerrimine/Jacobine	9.26	336.1806	0.2	C_18_H_25_NO_5_

**Table 2:** High-resolution mass spectrometric data and elemental composition of GD (negative ion mode).

No.	Component name	Retention time	Found at mass	Mass error (ppm)	Formula
1	Quinic acid	1.23	191.0562	0.5	C_7_H_12_O_6_
2	Uridine	2.23	243.0623	0.2	C_9_H_12_N_2_O_6_
3	Adenosine	2.44	266.0896	0.3	C_10_H_13_N_5_O_4_
4	Chlorogenic acid	4.97	353.0877	−0.3	C_16_H_18_O_9_
5	Caffeic acid	5.57	179.0351	0.8	C_9_H_8_O_4_
6	Methyl chlorogenate	6.31	367.1033	−0.3	C_17_H_20_O_9_
7	Isoferulic acid	7.32	193.0504	−1	C_10_H_10_O_4_
8	Kaempferol-3-*O*-rutinoside	7.53	593.151	−0.4	C_27_H_30_O_15_
9	Astragalin	7.87	447.093	−0.7	C_21_H_20_O_11_
10	Dicaffeoylquinic acid (Cynarin)	8.26	515.119	0	C_25_H_24_O_12_

### Effects of GD on the fasting blood glucose level of diabetic rats

**Fig. 4:**
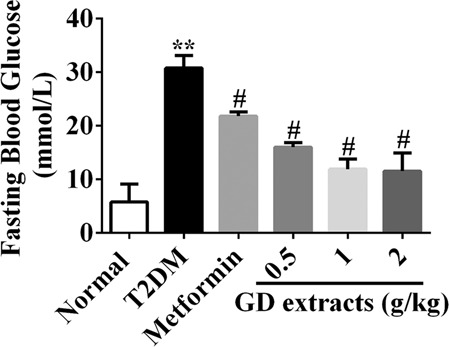
Effects of GD on fasting blood glucose level of rats. **P < 0.01 compared to the normal group; ^#^*P* < 0.05 compared to the T2DM group.

We treated T2DM model rats with GD for 4 weeks to test its anti-diabetic effects in vivo. As shown in Fig. 4, the FPG levels of the T2DM rats without GD treatment were significantly higher than those of the normal control rats (*P* < 0.05). After administration with GD for 4 weeks, the average FPG levels of the T2DM rats in the GD low-dose, mid-dose, and high-dose groups were 21.78, 21.37, and 21.34 mmol/L respectively, compared to 30.07 mmol/L in the model group, suggesting that GD significantly lowered the FPG level of the T2DM rats (*P* < 0.05). Metformin had a similar effect as GD.

### Effects of GD on water intake, food intake, and urine volume of diabetic rats

**Fig. 5:**
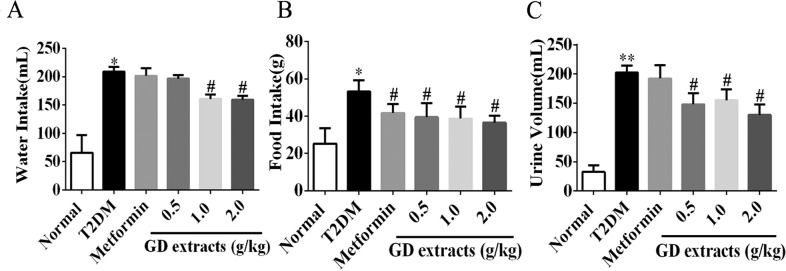
Effects of GD on water intake, food intake, and urine volume of diabetic rats. Effects of GD on water intake (**a**), food intake (**b**) and urine volume (**c**) of rats. *P < 0.05; **P < 0.01 compared to the normal group; ^#^*P* < 0.05 compared to the T2DM group.

Increased drinking, eating, and urination are common symptoms of diabetes^14,15^. Compared to the T2DM group, water intake, food intake, and urine output were significantly decreased in rats in the three GD groups (Fig. 5a–c, *P* < 0.05). These results suggest that GD can provide relief from diabetic symptoms. Metformin had almost no effect except that it reduced food intake to the same degree of GD.

### Effects of GD on biochemical parameters in diabetic rats

**Fig. 6:**
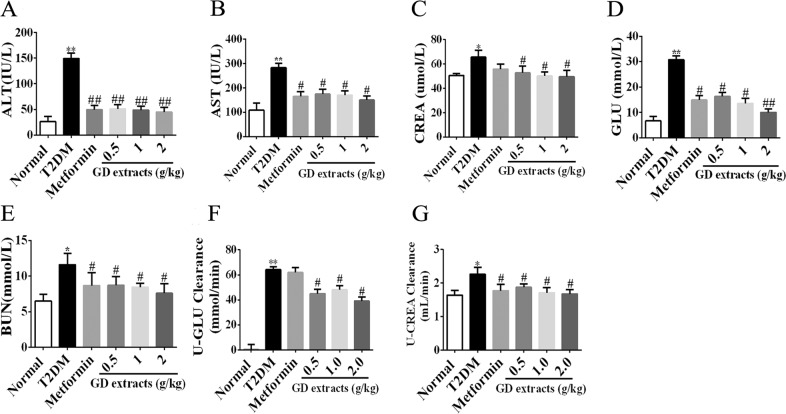
Effects of GD on biochemical parameters in diabetic rats. Effects of GD on biochemical parameters ALT (**a**), AST (**b**), CREA (**c**), GLU (**d**), BUN (**e**), U-GLU (**f**), and U-CREA (**g**) in rats. *P < 0.05; **P < 0.01 compared to the normal group; ^#^*P* < 0.05 compared to the T2DM group; ^##^*P* < 0.01 compared to the T2DM group.

Before the end of the study, blood and urine samples of the rats were collected for investigation of biochemical indicators, including ALT, AST, GLU, BUN, and CREA. As shown in Fig. 6a–e, the levels of these indicators in the metformin group and the three GD groups were significantly decreased compared to those in the model group (*P* < 0.05).

GLU and CREA in urine were also measured in the rats. U-GLU excretion volume and U-CREA clearance rate were calculated. As shown in Fig. 6f, U-GLU excretion was at 24 h significantly reduced in the three GD group compared to the T2DM group (*P* < 0.05). Moreover, the U-CREA clearance rate (Fig. 6g) in the GD groups was also significantly decreased (*P* < 0.05) compared to the T2DM group. These results showed that GD has potential protective effects on the rats’ liver and kidney. Metformin had similar but generally less profound effects as GD.

### Effects of GD on the PI3K/AKT signaling pathway and fatty acid metabolism signaling pathway

**Fig. 7:**
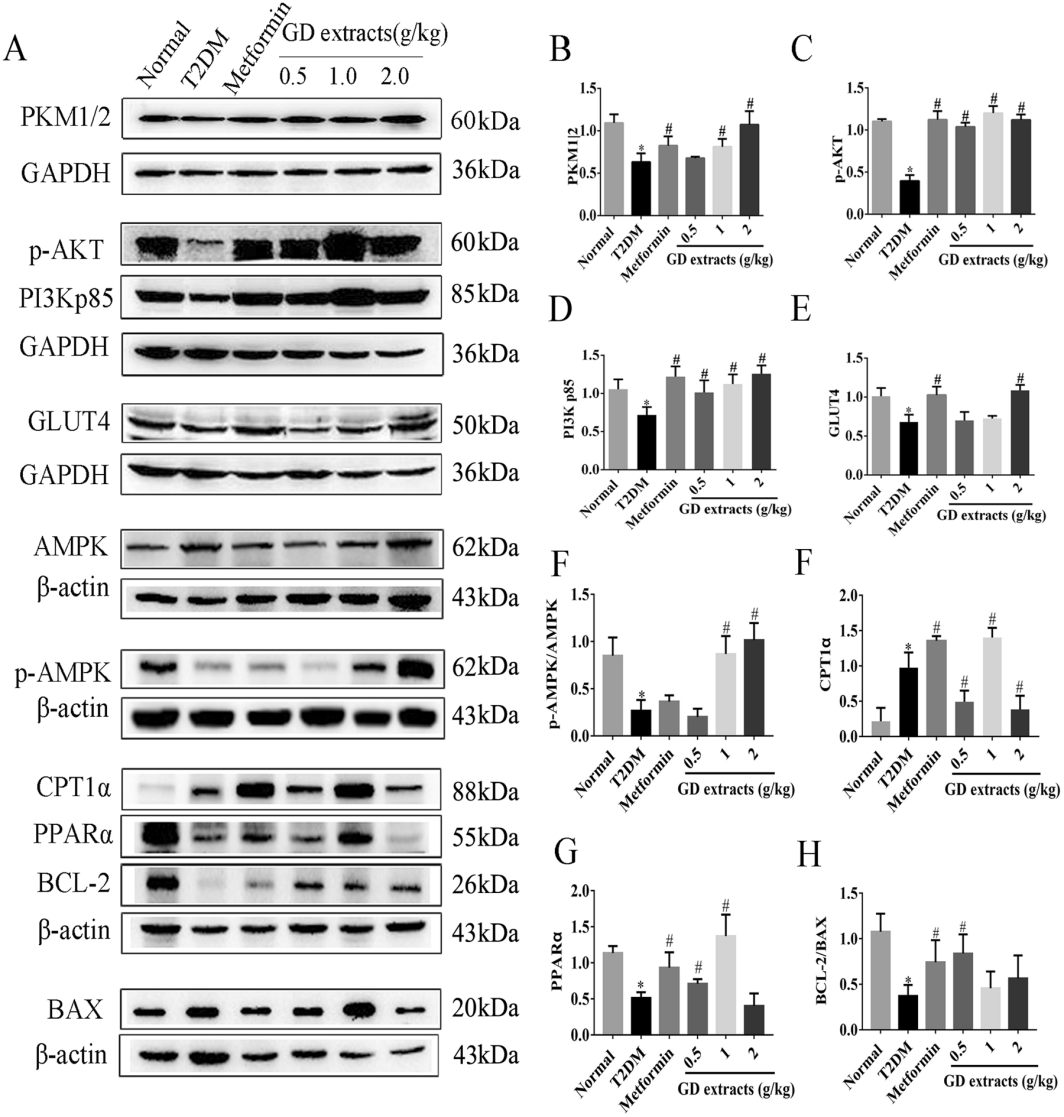
Effects of GD on the expression levels of key proteins in the PI3K/AKT signaling pathway and the fatty acid metabolism signaling pathway. **a** The protein levels of PKM1/2, p-AKT, PI3K p85, GLUT4, AMPK, p-AMPK, PPARα, CPT1α, BCL-2, and BAX in the rat liver from different groups were detected by immunoblotting. **b**–**h** The grayscale value analysis is represented on the bar graphs. *P < 0.05 compared to the normal group; ^#^*P* < 0.05 compared to the T2DM group.

To further elucidate the anti-diabetic mechanisms of GD, the expression levels of key proteins in the PI3K/AKT signaling pathway and fatty acid metabolism signaling pathway in the rat liver were analyzed by immunoblotting. As shown in (Fig. 7a–i), the expression levels of PKM1/2, p-AKT, PI3K p85, and GLUT4 in the PI3K/AKT signaling pathway were significantly upregulated. Moreover, the key proteins’ expression levels in the fatty acid metabolism signaling pathway including AMPK, p-AMPK, PPARα, and CPT1α were also significantly upregulated (*P* < 0.05). The anti-apoptotic gene BCL-2/BAX ratio was significantly upregulated after GD treatment. These data suggested that GD could activate AMPK, upregulate the PPARα pathway to promote the expression of CPT1α, and activate the PI3K/AKT signaling pathway to promote GLUT4 transport, thereby promoting the catabolism of fatty acids, reducing the blood glucose level, improving diabetic symptoms, and suppressing and protecting the liver and kidneys.

## Discussion

Diabetes is a complex disease characterized by disorders of glucose metabolism. In recent years, the incidence of diabetes has shown a rapid upward trend accompanying the improvement of living standards^16,17^. GD, one of the well-known anti-diabetic herbal medicines in China, was chosen to investigate its anti-diabetic effects and mechanisms in T2DM rats in this study.

PharmMapper is a web-based pharmacology platform for analyzing pharmacophore matching and potential drug targets^18^. The DAVID database provides gene annotation and signal pathway analysis for identifying biological functions of genes^19^. We have obtained 15 active ingredients of GD through extensive literature review. Active components obtained by UHPLC-Q/TOF-MS analysis were mostly consistent with the 15 reported compounds. Then 40 possible target genes of GD were predicted using PharmMapper, and gene annotation was finally identified using the DAVID database. Through the analysis of KEGG pathway annotation of STRING, the main pathway distribution of 10 potential targets, including metabolic pathways, insulin resistance, insulin signaling pathway, PPAR signaling pathway, bile secretion, purine metabolism, etc., have been identified. Next, we carried out a series of experiments to substantiate the bioinformatics results.

The liver is considered the center of metabolism, and it plays critical roles in the metabolism of matters and energy^20^. Many molecular metabolic processes, including the metabolism of carbohydrates, lipids, proteins, and hormones, are controlled by the liver^21^. Upon insulin stimulation, the liver is one of the major organs responsible for regulating the blood glucose level. Therefore, we chose the liver as the target organ for analyzing the anti-diabetic effects of GD.

Relative or absolute lack of insulin, reduced utilization of blood glucose in the liver and muscle tissues, and excessive output of hepatic glycogen are important causes of hyperglycemia in diabetes^22^. The liver is an essential organ responsible for glucose metabolism^23^. In diabetes, the glucose metabolism function of the liver is severely impaired^24^. ALT and AST are the most widely used indicators of hepatic injury^25^. ALT exists only in the cytoplasm, while AST is mainly found in the mitochondria. When hepatocytes are damaged, a large amount of ALT escapes from the cells. When hepatocytes are severely necrotic, AST releases from the mitochondria^26,27^. In this study, GD treatment in type 2 diabetic rats significantly reduced the serum levels of ALT and AST, indicating that GD can effectively protect the liver of diabetic rats.

Hyperglycemia causes severe damages to the kidneys and even induces severe diabetes kidney disease^28^. BUN and CREA are two important indices to reflect the degree of kidney damage^29^. In the blood biochemical index analysis, the levels of CREA and BUN of the T2DM group were significantly higher than those of the normal group, indicating that there was severe kidney damage in the diabetic rats. GD treatment significantly reduced the CREA and BUN levels of the T2DM rats. Therefore, GD also shows good protective effects toward the kidneys.

PKM1/2, PI3K, p-AKT, and GLUT4 are key players in the insulin signaling pathway in diabetes^30–32^. Activation of the PI3K/AKT signaling pathway inhibits high blood glucose-induced apoptosis and senescence in nucleus pulposus cells^33^. PKM1/2 is involved in glycolysis, which is closely related to the occurrence of diabetes^34,35^. GLUT4 is a glucose carrier/transporter located on the cell membrane^36^. A number of literature have reported that the increase of GLUT4 expression promotes the uptake and utilization of glucose by cells, thereby playing an integral role in the treatment of diabetes^37,38^. Our study demonstrated that GD upregulates the expression levels of PKM1/2, PI3Kp85, p-AKT, and GLUT4 and hence reducing the blood glucose level, improving diabetic symptoms, and protecting the liver and kidneys.

AMPK, p-AMPK, PPARα, and CPT1α are the key nodes of the fatty acid metabolism signaling pathway and are closely related to the occurrence of diabetes^39–41^. AMPK is a key regulator of energy metabolism and is abundantly expressed in metabolically related tissues. What is more, the activation of AMPK promotes oxidation of fatty acid beta^42,43^ and phosphorylation of AMPK could activate a large number of downstream target proteins, inhibit the synthesis of fat and cholesterol, and promote fatty acid oxidation and glucose transport^44^. It has been demonstrated that activation of AMPK directly promotes GLUT4 expression in the skeletal muscle^45^. In our study, T2DM rats’ blood glucose levels increased, while AMPK phosphorylation was reduced. On the other hand, the blood glucose levels of the GD-treated rats decreased, while the protein level of p-AMPK was significantly increased compared to the T2DM rats. These results suggest that GD could effectively provide relief from diabetic symptoms.

PPARα is a key regulator of lipid metabolism in the liver, and it regulates the transcription of the fatty acid oxidase gene and genes regulating cholesterol metabolism^46^. Studies have shown that AMPK could maintain liver lipid metabolism balance through PPARα. CPT1α is widely expressed in the liver, kidney, and adipose tissue. And it catalyzes the transfer of long-chain fatty acids into mitochondria for oxidative decomposition, maintaining the balance of blood sugar and energy supply^47^. Our experiments demonstrated that GD could promote the expression of PPARα and upregulate the expression of CPT1α to stimulate the oxidation of fatty acids. In addition, BCL-2 is an anti-apoptotic gene, which is closely related to liver damages^48^. BAX, a pro-apoptotic gene, belongs to the BCL-2 family that induces apoptosis^49^. The anti-apoptotic gene BCL-2/BAX protein ratio in rats was significantly upregulated after GD intervention. All results indicated that GD might have good effects of anti-apoptosis and protect hepatocytes.

PPAR nuclear receptor family as transcription factors regulate signaling of lipid metabolism and inflammation. Among them, PPARα is expressed predominantly in the liver, where it promotes fatty acid β-oxidation, lipid transport, and gluconeogenesis^50^. The PPARα agonist can provide relief from insulin resistance and hepatic steatosis in high-fat-diet-fed mice^51^. PPARα knockout mice will not express the proteins responsible for fatty acid metabolism^52^. It often forms heterodimers with RXR and enters the nucleus to regulate transcription by binding with the target genes such as fatty acid oxidase gene and cholesterol metabolism gene, which contained CPT1, SREBP1c, LXRα, and so on^53^. It has been shown that the level of PPARα, which positively regulates CPT1 and LXRα expression, is reduced in diabetes^54^.

And GLUT4, which is involved in AMPK pathway and PI3K/AKT pathway, can enhance insulin sensitivity and glucose tolerance. Activation of AMPK leads to an increase of GLUT4 expression^55^. However, further research is needed to determine whether AMPK can influence the PI3K/AKT pathway.

Our results showed that PPARα serves as a transcription factor to promote the expression of CPT1α and activate the PI3K/AKT signaling pathway to promote GLUT4 transport. These findings indicated that GD could promote the catabolism of fatty acids, reduce the blood glucose level, improve diabetic symptoms, and protect the liver and kidneys by avoiding against apoptosis. These results validated our network pharmacology analysis. Therefore, our study suggested that GD could be a good candidate for the development of effective hypoglycemic drugs to provide relief from diabetic symptoms.

## Conclusion

In summary, our study demonstrates that GD could significantly provide relief from diabetic symptoms in T2DM rats by regulating PI3K/AKT signaling and fatty acid metabolism.

## Supplementary information

Figure and Table Legends
