# Supplementary material for: Gynura divaricata exerts hypoglycemic effects by regulating the PI3K/AKT signaling pathway and fatty acid metabolism signaling pathway
Source: Nutr Diabetes. 2020 Aug 14;10:31. doi: 10.1038/s41387-020-00134-z (PMC7427804; doi:10.1038/s41387-020-00134-z)
Supplement: Supplementary file 1 — Figure and Table Legends [file 41387_2020_134_MOESM1_ESM.docx]

**Figure and Table Legends**

**Fig. 1** **Molecular structures of the 15 major active components in GD.** (A) Otosenine (C_19_H_27_NO_7_); (B) Sineciphyllinine (C_20_H_25_NO_6_); (C) Senkirkine (C_19_H_27_NO_6_); (D) Jacobine (C_18_H_25_NO_5_); (E) Integerrimine (C_18_H_25_NO_5_); (F) Uridine (C_9_H_12_N_2_O_6_); (G) Adenosine (C_10_H_13_N_5_O_4_); (H) Quercetin (C_15_H_10_O_7_); (I) β-sitosterol (C_29_H_50_O); (J) Kaempferol (C_15_H_10_O_6_); (K) Astragalin (C_21_H_20_O_11_); (L) nicotiflorin (Kaempferol-3-O-rutinoside) (C_27_H_30_O_15_); (M) methyl chlorogenate (C_17_H_20_O_9_); (N) kaempferol-3,7-di-O-p-D-glucoside (C_27_H_30_O_16_); (O) Kaempferol-5-O-(6′′-O-acetyl)-β-D-glucoside (C_23_H_22_O_11_).

**Fig. 2 GO enrichment of the GD potential target genes and KEGG pathway analysis results.** (A) Biological Process; (B) Molecular Function; (C) Cellular Component; (D) KEGG pathway.

**Fig. 3** **The total ion chromatogram of GD by UHPLC-Q/TOF-MS.** (A) positive ion mode, (B) negative ion mode.

**Fig. 4** **Effects of GD on fasting blood glucose level of rats.** ***, P < 0.01* compared to the normal group; *#, P < 0.05* compared to the T2DM group.

**Fig. 5 Effects of GD on water intake (A), food intake (B) and urine volume (C) of rats.** **, P < 0.05*; ***, P < 0.01* compared to the normal group; *#, P < 0.05* compared to the T2DM group.

**Fig. 6** **Effects of GD on biochemical parameters ALT (A), AST (B), CREA (C),GLU (D), BUN (E), U-GLU (F) and U-CREA (G) in rats.** **, P < 0.05*; ***, P < 0.01* compared to the normal group; *#, P < 0.05* compared to the T2DM group; ##, *P <* *0.01* compared to the T2DM group.

**Fig. 7 Effects of GD on the expression levels of key proteins in the PI3K/AKT signaling pathway and the fatty acid metabolism signaling pathway.** (A) The protein levels of PKM1/2, p-AKT, PI3K p85, GLUT4, AMPK, p-AMPK, PPARα, CPT1α, BCL-2 and BAX in the rat liver from different groups were detected by immunoblotting. (B-H) The grayscale value analysis was represented on the bar graphs. **, P < 0.05* compared to the normal group; *#, P < 0.05* compared to the T2DM group.

**Table 1** High resolution mass spectrometry data and elemental composition of GD (Positive Ion Mode).

**Table 2** High resolution mass spectrometry data and elemental composition of GD (Negative Ion Mode).
